# Effects of Algal-Derived *β*-Glucan on the Growth Performance, Intestinal Health, and *Aeromonas veronii* Resistance of Ricefield Eel (*Monopterus albus*)

**DOI:** 10.1155/anu/8172810

**Published:** 2025-01-21

**Authors:** Yu Guo, Zijing Yuan, Yueyun Han, Daiqin Yang, Hanwen Yuan, Fuxian Zhang

**Affiliations:** College of Animal Science and Technology, Yangtze University, Jingzhou 434023, China

**Keywords:** *β*-glucan, *Aeromonas verinii*, growth, intestinal flora, *Monopterus albus*

## Abstract

Ricefield eel is an important economic fish in China. However, large-scale intensive breeding has increased the incidence of diseases in eels. In this study, we conducted an 8-week feeding trial to investigate the effects of *β*-glucan on the growth performance, intestinal health, and *Aeromonas veronii* resistance of *Monopterus albus* (*M. albus*). Three hundred healthy fish (initial body weight: 12.38 ± 0.50 g) were randomly divided into five groups: A1 (basal diet) was considered the control group, whereas A2, A3, A4, and A5 were the experimental groups. The fish in the experimental groups were fed a basal diet supplemented with 250, 500, 1000, and 2000 mg/kg *β*-glucan, respectively. The addition of 0.025%–0.2% *β*-glucan resulted in a notable enhancement of eel growth performance, with the most significant improvement observed in eels supplemented with 0.1% *β*-glucan (*p* < 0.05). Furthermore, 0.025%–0.2% *β*-glucan could significantly enhance the antioxidant properties of the eel intestinal tract (*p* < 0.05), and the addition of 0.1% *β*-glucan significantly improved trypsin (TPS), amylase (AMS), and lipase (LPS) activities in the intestine (*p* < 0.05). In terms of intestinal histology, the A3, A4, and A5 groups exhibited significantly greater villus height compared to the control group (*p* < 0.05). Concentrations of *β*-glucan at 0.1% and 0.2% enhanced the composition of the intestinal flora; specifically, the relative abundance of Proteobacteria increased, while the relative abundance of Firmicutes decreased. Moreover, the addition of 0.05%–0.2% *β*-glucan significantly improved the relative survival rate (SR) of *A. veronii*-infected eels and significantly decreased the bacterial load of the liver, spleen, and kidney (*p* < 0.05). In comparison to eels that did not receive *β*-glucan supplementation, eels supplemented with 0.2% *β*-glucan exhibited decreased intestinal structural damage. In summary, the addition of 0.1%–0.2% *β*-glucan can promote eel growth, improve intestinal digestion and antioxidant capacity, regulate intestinal flora, and enhance intestinal physical function and anti-infection ability.

## 1. Introduction

Ricefield eel (*Monopterus albus*) is a freshwater cave-dwelling fish of the class Actinopterygii that is primarily distributed in Asian countries [1]. In China, eel is primarily distributed in the lower reaches of the Yellow River and the middle and lower reaches of the Yangtze River. This fish is a consumer favorite owing to its delicious taste and various nutrition [2]. The eel farming industry began in the 1980s, and innovations in aquaculture technology have led to rapid development and expansion of the aquaculture sector [3]. However, recently, increased breeding density and water pollution have contributed to a rise in disease incidence among eels, resulting in significant losses for the eel breeding industry [4]. The usage of antibiotics to prevent and control aquatic diseases has led to several problems, including water pollution, drug resistance, and drug residue. Therefore, its application is widely limited and seriously affects the healthy development of the eel industry [5]. Hence, discovering approaches to replace antibiotics is urgently warranted to improve the disease resistance of eel and realize the sustainable and healthy development of the eel breeding industry [6].

Studies have revealed that replacing antibiotics with plants or herbs for disease prevention can not only improve disease resistance and immunity but also promote the growth performance of fish [7, 8]. For example, the addition of 0.04% magnolol to tilapia feed significantly improved the antioxidant capacity and immune function of this fish [9]. Furthermore, the addition of 0.1% magnolol exhibited the best effect on promoting growth and anti-*Vibrio harvecii* infection [10]. Lastly, 1% of FCHM improved the growth performance, liver antioxidant capacity, and immune capacity of juvenile largemouth bass [11].

*β*-Glucan is a polysaccharide compound with a complex structure [12]. It is primarily divided into two sources, namely, cereal and noncereal sources, with different sources having different functions [13, 14]. Owing to the development of modern biotechnology, *β*-glucan is mostly extracted from algae [15]. *β*-Glucan is regarded as a promising immunostimulant, with results varying when incorporated into different animal feeds [16]. Research has shown that the inclusion of *β*-glucan in pig feed enhances growth performance and intestinal morphology in piglets while also increasing the population of beneficial bacteria in the intestine [17]. The addition of *β*-glucan to chicken feed not only improves growth performance but also alters the intestinal flora structure and reduces tumor necrosis factor-*α* concentration [18]. In aquaculture, *β*-glucan has been demonstrated to promote growth, enhance intestinal health, and regulate immunity in certain fish species [19–22]; however, relevant findings regarding eels remain unreported. Such as existing studies have revealed that the addition of 0.09% *β*-glucan can significantly promote the growth and nonspecific immune response of *Larimichthys crocea* [23]; researchers have noted that *β*-glucan can promote the intestinal villus growth of tilapia and expand the microbial colonization area [24]. In addition, oral *β*-glucan administration activates a nonspecific immune response in largemouth bass and increases resistance to *Aeromonas schubertii* [25].

Based on these findings, in the present study, we investigated the effects of algal-derived *β*-glucan on growth performance, intestinal health, and anti-*Aeromonas verinii* species in feed. Our results provide new insights into the application of *β*-glucan in the healthy breeding diet of eels.

## 2. Materials and Methods

### 2.1. Material Source

*β*-Glucan was derived from euglena powder, which contains 70.1% *β*-glucan, 22.2% protein, 3.4% fat, 1.5% moisture, and 2.8% ash.

Eels were purchased from an eel breeding base in Chibi City, Hubei Province. The Laboratory of Biology of Important Pathogens in Animals (College of Animal Science, Yangtze University) isolated *Aeromonas veronii*.

### 2.2. Experimental Diets

Eels were provided with a basal diet that met their growth and comprehensive nutritional needs. Fish meal and soybean meal served as the primary protein sources in this trial, while soybean oil and soybean lecithin were utilized as the main lipid sources, with wheat flour being the primary carbohydrate source. Detailed formulations are presented in Table 1. The control group was fed a basal diet supplemented with 0 mg/kg *β*-glucan (control group, A1), and the other four groups were fed with the basal diet supplemented with 250 (A2), 500 (A3), 1000 (A4), and 2000 (A5) mg/kg *β*-glucan, respectively [26]. The basal pellet feed was ground into a powder, to which the corresponding *β*-glucan was added for each group; the final product remained in powder form. After thorough mixing, 20% water was incorporated into the diets to form ball-shaped dough [27].

### 2.3. Experimental Procedure

All feeding experiments were conducted at the aquaculture base of Yangtze University. The water for cultivation was fully aerated pond water with natural light. Initially, the eels were temporarily housed in boxes located in a greenhouse for 1 week. Subsequently, 300 healthy eels (12.38 ± 0.50 g) were selected and distributed among 15 indoor plastic boxes (length, width, and height = 105 cm, 75 cm, and 50 cm, respectively). Five treatment groups were established, each consisting of three boxes, with 20 eels allocated per box. The eels in the control group were fed the basal diet supplemented with 0 mg/kg (A1) *β*-glucan, and the other four groups were fed the basal diet supplemented with 250 (A2), 500 (A3), 1000 (A4), and 2000 (A5) mg/kg *β*-glucan. The testing period lasted 8 weeks (2023.8.20–2023.10.20). Four eel nests were placed into each plastic box as shelter.

During the rearing period, the eels were fed once daily (at 18:00) at a rate of 2%–5% of their body weight. The prepared dough was placed on a floating net for the eels to consume. Daily observations of feed residue were conducted, and feeding amounts were adjusted accordingly. The water level during the test was maintained at 30 cm, with two-thirds of the water volume being changed every 2 days. The residual bait and feces were cleaned at regular intervals every day. The feeding situation and breeding water temperature were recorded. The breeding conditions of the temporary breeding and formal experiments were consistent: water temperature: 25–32°C, dissolved oxygen: 4.0 ± 0.3 mg/L, pH: 6.5–7.6, ammonia nitrogen: 0.060 ± 0.002 mg/L, and nitrite: 0.040 ± 0.005 mg/L.

### 2.4. Sample Collection

Following feeding, the eels were subjected to a 24-h starvation period. The number of eels in each box was then counted, and 15 eels were randomly selected from each group. The selected eels were anesthetized using MS-222, and their body weight and length were measured to calculate the weight gain rate (WGR), specific growth rate (SGR), survival rate (SR), and feed conversion ratio (FCR).

After dissection, the viscera and liver weight were determined and condition factor (CF), hepatosomatic index (HSI), and viscerosomatic index (VSI) were calculated. The intestines were isolated during dissection to determine digestive enzyme activity and antioxidant properties and detect intestinal flora. Thirty fish from each experimental group were subsequently used in the challenge experiment. They were fed the same feed as the experimental group.

### 2.5. Growth Performance

The following formulas were used to calculate the growth performance parameters:

  $$\begin{array}{c} \text{Survival rate }\left(\text{SR},\%\right)=100\%\times \left(\text{Final number of fish}\right)/\left(\text{initial number of fish}\right), \end{array}$$

  $$\begin{array}{c} \text{Weight gain rate }\left(\text{WGR},\%\right)=100\%\times \left(\text{Final body weight }\left(g\right)-\text{initial body weight }\left(g\right)\right)/\left(\text{initial body weight }\left(g\right)\right), \end{array}$$

  $$\begin{array}{c} \text{Specific growth rate }\left(\text{SGR},\%/\text{day}\right)=100\%\times \left(\text{Ln }\left(\text{final body weight}\right)-\text{Ln }\left(\text{initial body weight}\right)\right)/\left(\text{the duration of the experiment }\left(\text{days}\right)\right), \end{array}$$

  $$\begin{array}{c} \text{Condition factor }\left(\text{CF},g/{\left(\text{cm}\right)}^{3}\right)=100\%\times \left(\text{Body weight }\left(g\right)\right)/\left(\text{body length }{\left(\text{cm}\right)}^{3}\right), \end{array}$$

  $$\begin{array}{c} \text{Viscerosomatic index }\left(\text{VSI},\%\right)=100\%\times \left(\text{Viscera weight }\left(g\right)\right)/\left(\text{whole body weight }\left(g\right)\right), \end{array}$$

  $$\begin{array}{c} \text{Hepatosomatic index }\left(\text{HSI},\%\right)=100\%\times \left(\text{Liver weight }\left(g\right)\right)/\left(\text{whole body weight }\left(g\right)\right). \end{array}$$

### 2.6. Enzyme Activity Analysis

The intestinal tissue was suspended in precooled saline at a mass-to-volume ratio of 1:9, shaken by ultrasound, centrifuged at 4000 g and 4°C for 10 min, and the supernatant was taken for determination. The activities of intestinal digestive enzymes, including amylase (AMS), trypsin (TPS), and lipase (LPS), as well as indices related to intestinal antioxidant capacity, such as superoxide dismutase (SOD), malondialdehyde (MDA), and catalase (CAT), were determined using colorimetric methods. Commercial assay kits (Jiancheng Bioengineering Institute, Nanjing, China) were used according to the manufacturer's instructions to determine enzyme activities.

### 2.7. Intestinal Morphology

Three fresh fish intestines from each group were fixed in a 4% paraformaldehyde solution and subsequently embedded in paraffin. Fixed foregut samples were dehydrated with 55%–90% ethanol, followed by the preparation of tissue sections. These sections were stained with hematoxylin and eosin (H&E), and then observed and photographed them under a microscopeanalyze intestinal morphological parameters. Image J software was used to measure the length of the foregut villus.

### 2.8. Intestinal Microflora Analysis

Library sequencing: After total DNA extraction from the sample, primers F (ACTCCTACGGGAGGCAGCA) and R (GGACTACHVGGGTWTCTAAT) were obtained based on the design of the conservative region. A sequencing connector was added to the primer end, followed by PCR amplification. The products were purified, quantified, and made homogeneous to generate the sequencing library. The Illumina NovaSeq 6000 platform was used to sequence the qualified library. Base calling was used to convert the original image data files obtained via high-throughput sequencing (Illumina NovaSeq and other sequencing platforms) into the original sequence reads.

Quality filtering: The sequenced raw reads were filtered using Trimmomatic v 0.33 software. Then, cutadapt 1.9.1 software was used to identify and remove the primer sequences to obtain clean reads without the primer sequence.

DADA2 denoising: Denoising was performed using QIIME2 2020.6. Double-ended sequences were spliced, and chimeric sequences were removed to obtain the final valid data.

### 2.9. Challenge Experiment

*A. veronii* was inoculated into 5 mL of liquid trypticase soy broth medium and cultured at 28°C for 24 h. Then, they were centrifuged at 4200 × *g* for 5 min. The supernatant was discarded, and the pelleted bacteria were washed two times with sterile phosphate-buffered saline (PBS, pH 7.2). A spectrophotometer was used to adjust the *A. veronii* suspension to 2.1 × 10^11^ CFU mL^−1^, corresponding to the median lethal dose previously determined in a preliminary test. After 24 h of feeding trial sampling, 15 fish were randomly selected from each tank and intraperitoneally injected with 0.2 mL of the bacterial suspension using 27-gauge needles. During the 7-day challenge period, the fish were fed the respective diets and observed to record any abnormal behavior and mortality.

The fish were removed when they died. GraphPad Prism 9.0 was used to perform survival analysis. The fish samples were dissected, and the internal organs were separated. Then, 1 g of the sample was added to 1 mL of PBS, followed by grinding with a tissue homogenizer. The samples were diluted to different multiples before coating the culture dish. These culture dishes were incubated overnight at 28°C, followed by counting and analysis using GraphPad Prism 9.0. Fixed mid-gut and spleen samples were dehydrated with 55%–90% ethanol to prepare tissue sections to assess the morphological parameters of the intestine and spleen.

### 2.10. Statistical Analysis

The experimental results were expressed as means ± standard deviation (SD). One-way analysis of variance (ANOVA) and Student's *t*-test were conducted using GraphPad Prism 9.0 software to assess homoscedasticity and the normal distribution of all data. Duncan's multiple range test was applied for multiple comparisons between groups, with a significance level set at *p* < 0.05. All raw data were processed using Microsoft Excel 2016 (Microsoft, USA) and subsequently organized into three-line tables using Microsoft Word 2016 (Microsoft, USA) and figures created with GraphPad Prism 9.0 (GraphPad Software, USA).

## 3. Results

### 3.1. Growth Performance

Compared to the control group (A1), the FBW, WGR, SGR, and HSI were significantly elevated in the other four experimental groups (*p* < 0.05). Meanwhile, only the FCR exhibited a significant decreasing trend in response to dietary *β*-glucan (*p* < 0.05). The fish fed the A4 diet demonstrated the highest WGR and the lowest FCR when compared to the A1 group. However, the addition of *β*-glucan did not significantly influence SR, HSI, and CF (*p* > 0.05) (Table 2).

### 3.2. Intestinal Digestive Enzyme Activity

Regarding intestinal digestive enzymes, the TPS activity in the A3, A4, and A5 groups, as well as the AMS and LPS activity in the A4 group, were significantly higher than those in the A1 group (*p* < 0.05). Furthermore, the A4 diet significantly enhanced the activities of TPS, AMS, and LPS (*p* < 0.05) (Table 3).

### 3.3. Intestinal Antioxidant Capacity

**Figure 1:**
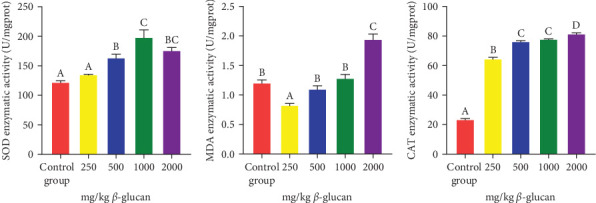
Antioxidant enzyme activities in *M. albus* were evaluated after 8 weeks of feeding diets supplemented with varying doses of *β*-glucan. The abscissa represents the group names, while the ordinate indicates the antioxidant activity. Standard deviations are shown with error bars, utilizing different colors to differentiate groups with varying *β*-glucan concentrations. Bars labeled with different letters indicate significant differences among treatments (*p* < 0.05). (a) Statistical plot of superoxide dismutase (SOD) activity. (b) Statistical plot of malondialdehyde (MDA) activity. (c) Statistical plot of catalase (CAT) activity.

In comparison to the A1 group, SOD activity was elevated in the A3, A4, and A5 groups (*p* < 0.05) (Figure 1a). Conversely, intestinal MDA activity was significantly reduced in group A2 and significantly increased in group A5 when compared to the A1 group (*p* < 0.05) (Figure 1b). Additionally, CAT activity related to intestinal antioxidant capacity was influenced by dietary *β*-glucan in a significant linear trend (*p* < 0.05), with the highest CAT activity observed in the A5 group (Figure 1c).

### 3.4. Foregut Morphology

**Figure 2:**
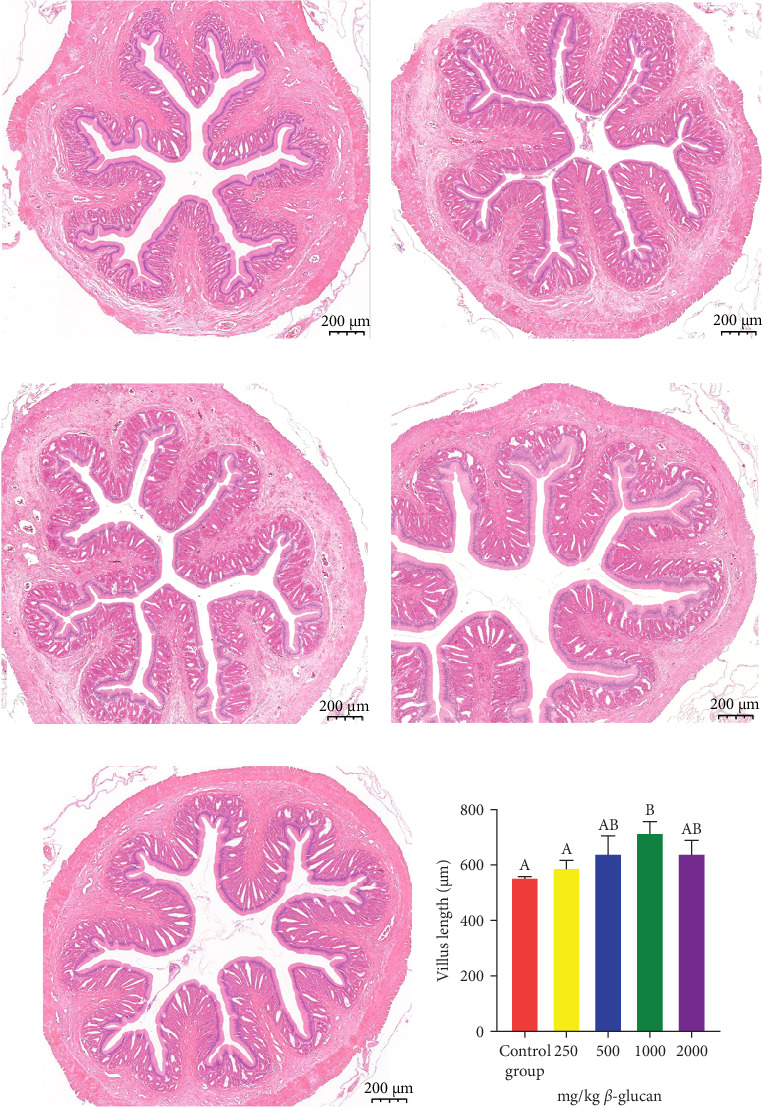
Effects of dietary *β*-glucan on the foregut morphology of *M. albus* are presented. (a) 0% (A1) *β*-glucan. (b) 0.025% (A2) *β*-glucan. (c) 0.05% (A3) *β*-glucan. (d) 0.1% (A4) *β*-glucan. (e) 0.2% (A5) *β*-glucan. (f) Length of intestinal villi. Scale bar: 200 µm. Bars labeled with different letters indicate significant differences among treatments (*p* < 0.05).

Figure 2 illustrates the intestinal tissue morphology of eels-fed diets with varying concentrations of *β*-glucan. The A3 (Figure 2c), A4 (Figure 2d), and A5 (Figure 2e) groups demonstrated a larger intestinal cross-sectional area and a greater number of intestinal villi compared to the control group. When compared to the A1 (Figure 2a) group, the length of intestinal villi in group A4 was significantly increased (*p* < 0.05), while no significant changes were observed in groups A2 (Figure 2b), A3, and A5 (*p* > 0.05).

### 3.5. Intestinal Microbiota Analysis

**Figure 3:**
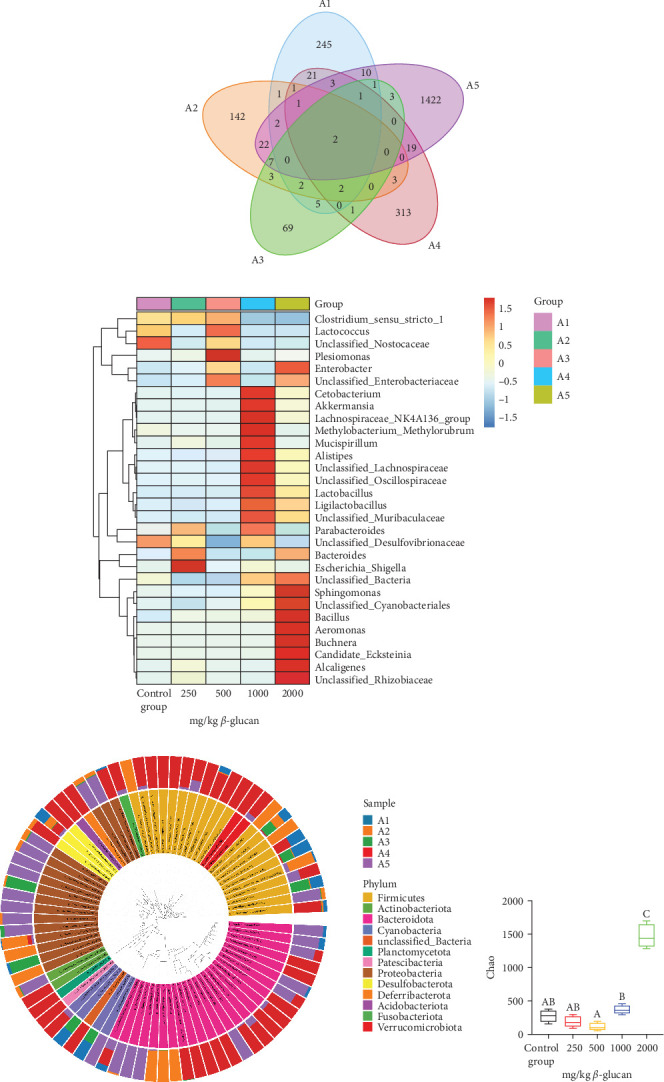
Analysis diagram of the intestinal flora. The abscissa represents the group name. (a) The ordinate represents the relative abundance of bacteria at the phylum level. Different colors are used to indicate different gates. The relative abundance of only the top 10 phyla is shown, with other species being combined as others. (b) The ordinate represents the relative abundance of bacteria at the genus level. Different colors indicate different gates. The relative abundance of only the top 10 genera is shown, with other species being combined as others. Unclassified represents species without taxonomic annotation. (c) Venn graph. Different colors are used to indicate different groups, and the numbers overlapping between different color figures represent the number of features shared between the two groups. (d) Community heatmap of species abundance clustering at the genus level. The ordinate is the species taxa: the cluster tree on the left represents the species cluster tree, whereas the top cluster tree is the sample cluster tree heat. Figure color gradient: The color shift from blue to red indicates the relative proportion of a species between different samples, from low to high. (e) Sample community distribution map of the species evolutionary tree. The upper right legend demonstrates the species name at the phylum level, the inner circle is the species evolutionary tree, and the same phylum is present in the inner circle species. The outer circle indicates the relative abundance ratio of the species in the different groups. (f–h) Box plot of the alpha diversity index. The index name is indicated in the ordinate, which is the index size. Bars labeled with different letters indicate significant differences among treatments (*p* < 0.05).

At the phylum level (Figure 3a), Firmicutes, Proteobacteria, Bacteroidota, Desulfobacterota, Actinobacteriota, and Cyanobacteria were identified as the dominant bacteria. The abundances of these five dominant phyla were 94.4%, 99.6%, 98.2%, 85.2%, and 84.7% in the A1, A2, A3, A4, and A5 groups, respectively. Compared to the A1 group, the proportion of Proteobacteria increased, while the proportion of Desulfobacterota decreased in the A2, A3, A4, and A5 groups.

At the genus level (Figure 3b), Clostridium was the dominant species in the A1, A2, and A3 groups. In the A4 group, the proportion of Methylobacterium increased, while the proportion of Clostridium decreased compared to the A1 group; in the A5 group, the proportion of Buchnera increased, and the proportion of Clostridium decreased.

The Venn diagram analyzed the similarities and differences of operational taxonomic units (OTU) among the groups (Figure 3c). A total of two common OTUs were identified across the five groups. We noted that the number of unique OTU in the eel intestinal flora was 245 in the A1 group, 142 in the A2 group, 69 in the A3 group, 313 in the A4 group, and 1422 in the A5 group.

Using a community heatmap, the microbial abundance of the top 30 genera in each group was analyzed (Figure 3d). Compared to the A1 group, the relative abundance of Escherichia increased in the A2 group, while the relative abundance of Plesiomonas increased in the A3 group. Notably, the abundance of eight bacterial species, including Cetobacterium, Akkermansia, Lachnospiraceae, Methylobacterium, Mucispirillum, Alistipes, unclassified_Lachnospiraceae, and unclassified_Oscillospiraceae, was elevated in the A4 group. Additionally, seven bacterial species, including Sphingomonas, Bacillus, Aeromonas, Buchnera, Candidatus, Alcaligenes, and unclassified_Rhizobiaceae, showed increased abundance in the A5 group.

The sample community distribution map of the species evolutionary tree provides information on the evolutionary relationships and relative abundance ratios between species (Figure 3e). Firmicutes, Proteobacteria, and Bacteroidota were identified as the dominant gut microflora in the A4 and A5 groups, with other strains also showing a higher abundance in both groups.

Gut microbial alpha diversity is illustrated in Figure 3f–h. The Chao index was significantly higher in the A5 group, while the Simpson and Shannon indices were significantly elevated in the A4 and A5 groups compared to the A1 group (*p* < 0.05). No significant differences were observed among the other groups (*p* > 0.05).

### 3.6. Challenge Test

**Figure 4:**
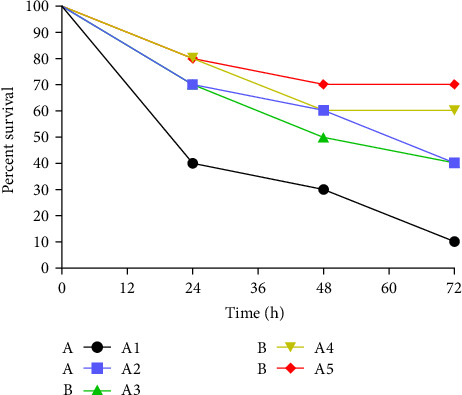
Survival rate of *A. veronii*-infected eel within 72 h.

After 72 h of infection with *A. veronii*, the SR of the A1 group was only 10%. In contrast, the SRs of the A2, A3, A4, and A5 groups were 40%, 40%, 60%, and 70%, respectively. After *A. veronii* infection, the SR of the A3, A4, and A5 groups is significantly higher than that of the control group (*p* < 0.05) (Figure 4).

**Figure 5:**
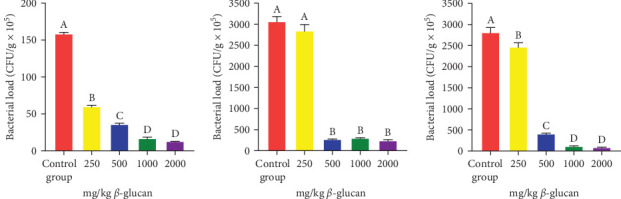
Organ bacterial load of each group at 24 h after the challenge. (a) Liver. (b) Spleen. (c) Kidney. Bars labeled with different letters indicate significant differences among treatments (*p* < 0.05).

After 24 h of the challenge, dead eels were removed, and the bacterial load of each tissue was determined. The number of *A. veronii* was significantly reduced in the spleen (Figure 5b) of the A3, A4, and A5 groups compared to the A1 group (*p* < 0.05). Furthermore, the bacterial load in the liver (Figure 5a) and kidney (Figure 5c) significantly decreased in the A2, A3, A4, and A5 groups (*p* < 0.05), with the A5 group exhibiting the lowest bacterial load in each organ.

**Figure 6:**
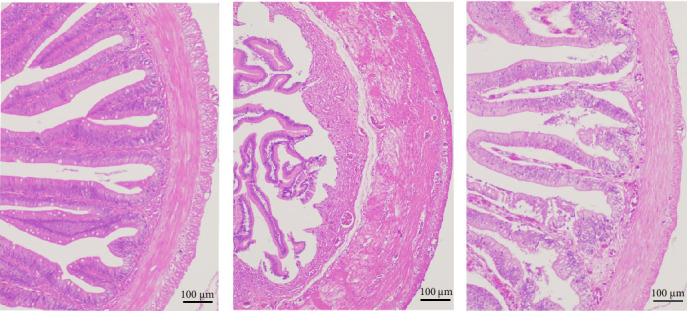
Comparison of eel tissue sections after challenge with *A. veronii*. (a) Healthy intestinal sections were observed in the A1 group. (b) The intestine of eels challenged with *A. veronii* intestinal in the A1 group. (c) The intestine of eels challenged with *A. veronii* in the A5 group. (a–c) 100× magnification. Scale bar: 100 µm.

Analysis of the intestinal sections of eels challenged with *A. veronii* indicated that, compared to healthy eels not challenged with *A. veronii* and not fed *β*-glucan (Figure 6a), the eels in the A1 group displayed severe intestinal villus fracture, muscle damage, and a dissolved mucosal layer (Figure 6b). In contrast, less cell shedding was observed in the epithelial cells of the challenged eels in the A5 group (Figure 6c).

## 4. Discussion

*β*-Glucan can improve the growth performance of aquatic organisms [28]. For example, Ji et al. [29] have reported that 0.2% *β*-glucan can significantly improve the WGR, SGR, and FCR of *Oncorhynchus mykiss*. Similarly, Lin et al. [30] have reported that *β*-glucan addition in the feed significantly increased the SGR of *Cyprinus carpio* koi. Furthermore, Ai et al. [23] have reported that 0.09% *β*-glucan addition significantly promoted the growth and nonspecific immunity of *L. crocea*. These findings are consistent with our findings that an increase in *β*-glucan concentration from 0.025% to 0.1% significantly increases the growth performance of eel, which significantly decreases with a further increase in *β*-glucan addition. This indicates that the addition of an appropriate amount can promote eel growth. Similar findings have been reported by Huang et al. [31] in aquatic organisms, where both sulfated and nonsulfated *β*-glucan from *Saccharomyces cerevisiae* (sGSC and GSC, respectively) promoted the growth of *Procambarus clarkii* compared with the control group. The highest weight and body length were noted in the sGSC 100 group. However, a further increase in sGSC concentration gradually diminished its promoting effect, with similar results in chickens [32]. This phenomenon may be because high *β*-glucan concentrations will inhibit the absorption of other substances, thereby decreasing the weight gain of eel. At present, the study results of the effects of *β*-glucan on the growth performance of aquatic animals remain controversial, and some studies have revealed that *β*-glucan does not promote the growth of aquatic animals, including *Oreochromis niloticus* [33], *Dicentrarchus labrax* [34], and *Ictalurus punctatus* [35]. These results suggest that the effect of *β*-glucan on the growth performance of aquatic animals is associated with the species, developmental stage, and its addition amount.

Fish growth is closely associated with the intestinal digestive and absorption capacities [36, 37], with intestinal digestive enzyme activity functioning as an indicator of the digestive and absorption capacities of nutrients [38]. Studies have revealed that *β*-glucan addition can significantly increase the activities of aminopeptidase, TPS, and chymotrypsin [39]. In our study, the dietary addition of *β*-glucan enhanced the activities of intestinal digestive enzymes in eels. However, the digestive enzyme activity began to decrease when the *β*-glucan concentration exceeded 1 g/kg. These findings suggest that the ability of *β*-glucan to improve intestinal digestion and absorption is parallel to an increase in weight gain. To alleviate oxidative stress, fish have evolved complete antioxidant enzyme systems (such as SOD, CAT, glutathione reductase, glutathione peroxidase, and glutathione S-transferase) to decrease redox molecules [40, 41]. *β*-Glucan addition enhanced the antioxidant capacity of the intestine, as manifested by a decrease in MDA content and an increase in SOD and CAT activities. Similarly, andrographolide can reduce oxidative stress damage in chondrocytes by enhancing antioxidant enzyme (SOD and CAT) activities in articular chondrocytes [42].

Intestinal microbes in aquatic animals are more mobile and sensitive to food changes [43]. In this study, the core bacteria in the gut were Firmicutes, followed by Proteobacteria and Bacteroidota. This finding is similar to that of previous studies on aquatic organisms such as *P. clarkii* [44], *Lateolabrax japonicus* [45], and *Euphausia pacifica* [46]. Contradicting the results of other researchers, *β*-glucan addition resulted in significant differences in the alpha diversity of the eel intestine [47]. This may be owing to the different breeding environments and test animals [43]. In addition, studies have demonstrated that high diversity and complex microbial communities contribute to the health of both human and animal hosts [48].

Reports indicate that Proteobacteria can withstand pollution and extreme environments [49]. Desulfobacterota typically have the ability to reduce sulfate as part of their metabolic processes, often leading to the production of hydrogen sulfide. Previous research has established associations between Desulfovibrio and various diseases, including intestinal inflammation, joint inflammation, and diabetes [50]. Additionally, oral administration of *Desulfovibrio desulfuricans* has been shown to exacerbate atherosclerotic lesions in mice, thereby increasing intestinal permeability and systemic inflammation [51]. In this study, it was observed at the phylum level that the addition of *β*-glucan increased the abundance of Proteobacteria and decreased the abundance of Desulfobacterota. This change may explain why the antioxidant and immune capacities of eels in the *β*-glucan group were superior to those in the non-*β*-glucan group. Furthermore, studies have analyzed genetic factors related to Methanobacteria through GWAS and concluded that Methanobacteria can promote poultry growth [52]. In this study, the abundance of Methanobacterium at the genus level significantly increased with the addition of 1000 mg/kg *β*-glucan. Simultaneously, the growth performance of this group of eels was the highest, which may be associated with Methanobacterium.

Bacterial regression experiments are widely performed to assess the health status of fish. *A. veronii*, a new type of human–fish co-infected opportunistic pathogen, is widely distributed in a large number of animals domestically and internationally, including eel [53]. Recently, this pathogen was isolated and identified in China, with the large-scale death of catfish in 2011 being a representative case. After isolating and identifying the pathogen, the pathogen was finally determined to be *A. veronii* [54]. In 2014, after the widespread death of California perch, the causative pathogen was isolated and identified as *A. veronii* [55]. However, studies on preventing *A. veronii* infection are limited, and most outbreaks were treated with antibiotics after onset. Our study is the first to explore the effect of *β*-glucan on *A. veronii* infection in eels. This is in line with the findings of Rørstad et al. that *β*-glucan significantly increases the resistance of *Salmo salar* against *A. hydrophila* [56]. Moreover, through challenge experiments, Sirimanapong et al. have reported that *β*-glucan increases the survival of *Pangasianodon hypophthalmus* [57]. The bacterial load directly reflects the severity of bacterial infection in fish [58]. The different SRs reflect the different degrees of resistance to *A. veronii* in eel fed with different *β*-glucan concentrations, which correlates with the number of pathogenic bacteria in the liver, spleen, and kidney tissues. In our study, *β*-glucan significantly decreased the bacterial load of the three tissues. Furthermore, histopathological slides can directly reflect tissue damage following bacterial infection [59]. In this study, intestinal and spleen slices directly reacted with *β*-glucan to protect the physical structure of tissues. However, additional studies are warranted to determine the mechanism by which *β*-glucan improves body immunity and how to improve the mechanism of immune tissues.

## 5. Conclusion

An appropriate amount of algal-derived *β*-glucan can improve the growth performance and intestinal health of eels. Furthermore, it can improve the activities of intestinal digestive and antioxidant enzymes and resistance to *A. veronii*. However, excessive *β*-glucan addition will decrease the growth performance and digestive enzyme activities of eels. Collectively, adding 0.1% *β*-glucan to the feed of eels is recommended. Although *β*-glucan is a natural, safe, and effective alternative to antibiotics, its mechanism of action warrants further investigation.

## Figures and Tables

**Table 1:** Formulation and proximate composition of the basal diet (dry weight).

Ingredients (%)	Composition
Fish meal	45
Soybean meal	12
Corn protein powder	5
Wheat flour	25
Compound protein^a^	2
Shrimp meal	1.5
Soybean oil	2
Soybean lecithin	2
Premix^b^	1
Earthworm meal	2
Dicalcium phosphate	2
Choline	0.3
Phytase	0.2
Proximate composition	—
Moisture	12.37
Crude protein	44.78
Crude lipid	7.92
Ash	11.64

^a^Complex protein is mainly formulated from soybean protein concentrate.

^b^The premix provided the following per kg of the diet: VD3, 2500.0 IU; VE, 200.0 mg; VK3, 10.0 mg; VB1, 25.0 mg; VB2, 45.0 mg; nicotinic acid, 200.0 mg; VB6, 20.0 mg; Ca-pantothenate acid, 60.0 mg; folic acid, 10.0 mg; VB12, 0.1 mg; biotin, 1.5 mg; VC, 200.0 mg; inositol, 200.0 mg; NaSeO_3_·5H_2_O, 0.3 mg; CoCZ·6H_2_O, 0.4 mg; KI, 0.8 mg; CuSO_4_·5H_2_O, 10.0 mg, MnSO_4_·4H_2_O, 20.0 mg; ZnSO_4_·H_2_O, 50.0 mg; FeSO_4_·7H_2_O, 150.0 mg; MgSO_4_·7H_2_O, 500.0 mg; NaCl, 1000.0 mg.

**Table 2:** Growth performance of *M. albus*-fed diets supplemented with different doses of *β*-glucan after 8 weeks.

Parameters	Diets (*β*-glucan %)				
	0.000 (A1)	0.025 (A2)	0.050 (A3)	0.100 (A4)	0.200 (A5)
IBW (g)	12.38 ± 0.50	12.38 ± 0.50	12.38 ± 0.50	12.38 ± 0.50	12.38 ± 0.50
FBW (g)	21.68 ± 0.78^a^	29.2 ± 0.68^bd^	30.44 ± 1.22^bd^	32.80 ± 0.87^c^	30.30 ± 0.75^d^
SR (%)	100 ± 0.00	100 ± 0.00	100 ± 0.00	100 ± 0.00	100 ± 0.00
WGR (%)	63.50 ± 5.91^a^	120.21 ± 5.11^bd^	130.02 ± 9.54^bd^	147.36 ± 6.57^c^	128.51 ± 5.65^bd^
SGR (%/day)	0.82 ± 0.06^a^	1.31 ± 0.04^bd^	1.39 ± 0.07^bd^	1.51 ± 0.04^c^	1.38 ± 0.04^bd^
FCR	2.63 ± 0.22^b^	1.38 ± 0.06^a^	1.28 ± 0.10^ad^	1.12 ± 0.05^c^	1.29 ± 0.06^ac^
HSI (%)	6.06 ± 0.84	6.17 ± 0.53	6.33 ± 1.23	6.96 ± 0.81	7.10 ± 1.02
VSI (%)	9.28 ± 0.88^a^	11.35 ± 1.02^bd^	11.12 ± 0.90^bd^	12.89 ± 0.93^c^	12.41 ± 1.28^cd^
CF (g/cm^3^)	0.10 ± 0.02	0.10 ± 0.01	0.08 ± 0.01	0.09 ± 0.01	0.08 ± 0.01

*Note:* No letter or the same letter indicates no significant difference (*p* > 0.05) and a significant difference (*p* < 0.05).

Abbreviations: CF, condition factor; FBW, final body weight; FCR, feed conversion ratio; HSI, hepatosomatic index; IBW, initial body weight; SGR, specific growth rate; SR, survival rate; VSI, viscerosomatic index; WGR, weight gain rate.

**Table 3:** Digestive enzyme activities in *M. albus*-fed diets supplemented with different *β*-glucan doses after 8 weeks.

Parameters	Diets (*β*-glucan%)				
	0.000 (A1)	0.025 (A2)	0.050 (A3)	0.100 (A4)	0.200 (A5)
TPS (U/mgprot)	1322.68 ± 62.10^a^	1500.74 ± 72.59^a^	2565.92 ± 118.31^bc^	2987.43 ± 37.1^b^	2385.36 ± 88.5^c^
AMS (U/mgport)	25.27 ± 1.01^a^	51.69 ± 3.27^a^	31.28 ± 3.30^ab^	37.88 ± 4.87^b^	25.27 ± 4.32^ac^
LPS (U/mgprot)	281.23 ± 3.17^a^	294.46 ± 0.85^a^	302.81 ± 0.84^a^	336.36 ± 6.62^b^	307.81 ± 9.92^ab^

*Note:* The same letter indicates no significant difference (*p* > 0.05) and a significant difference (*p* < 0.05).

Abbreviations: AMS, amylase; LPS, lipase; TPS, trypsin.

## Data Availability

The data can be available from the corresponding author upon reasonable request. The data are not publicly available due to privacy or ethical restrictions.
